# High-Density Fermentation of *Lactobacillus plantarum* P6: Enhancing Cell Viability via Sodium Alginate Enrichment

**DOI:** 10.3390/foods13213407

**Published:** 2024-10-25

**Authors:** Feiyang Sun, Siyi Liu, Xinying Che, Gang Wang, Xiufeng Wang, Yanli Li, Sitong Zhang, Huan Chen

**Affiliations:** 1 College of Life Science, Jilin Agricultural University, Changchun 130118, China; 13596173554@163.com (F.S.); siyiliu@mails.jlau.edu.cn (S.L.); 18946784669@163.com (X.C.); ylli@jlau.edu.cn (Y.L.); stzhang@jlau.edu.cn (S.Z.); chjlau@163.com (H.C.); 2 Key Laboratory of Straw Comprehensive Utilization and Black Soil Conservation, Education Ministry of China, Changchun 130118, China; 3 Vegetable and Flower Science Research Institute of Jilin Province, Changchun 130119, China; mailwxf@126.com

**Keywords:** LAB, fermentation optimization, high-density fermentation, response surface analysis, total colony count

## Abstract

*Lactobacillus plantarum* exhibits a wide range of beneficial physiological functions, including maintaining intestinal microbiota balance, reducing serum cholesterol, and promoting digestive health. According to the specific nutrient requirements of *Lactobacillus plantarum* P6, we investigated the effects of various carbon sources, nitrogen sources, trace elements, growth-promoting substances, as well as the initial pH and inoculum size on the growth of *Lactobacillus plantarum* P6 under fermentation conditions. The optimal growth conditions for *Lactobacillus plantarum* P6 were identified to facilitate high-density fermentation in small-scale fermenter production, achieving a cell concentration of 1.03 × 10^11^ CFU/mL. This resulted in a 2.5-fold increase in bacterial wet weight, and fermentation time was reduced to 12 h when utilizing a specific medium enriched with 0.2% sodium alginate. It is hypothesized that sodium alginate forms a protective film around the bacterial cells, promoting cell aggregation and enhancing self-coalescence, potentially triggering a bacterial community effect. These results provide a basis for the industrial-scale high-density cultivation of *Lactobacillus plantarum*, offering potential for enhanced biotechnological applications.

## 1. Introduction

Lactic acid bacteria (LABs) are a group of live microorganisms that, when administered in sufficient quantities, confer significant health benefits to the host [1], making them a key category of probiotics. They possess a wide range of biological activities, including modulating the composition of the gut microbiota, enhancing immune function, increasing resistance to pathogens, mitigating allergic responses [2,3], reducing serum cholesterol levels [4], and exhibiting antiviral effects [5]. These multifaceted properties have led to their extensive application in various industries, including food production, pharmaceuticals, and animal feed.

For instance, *Lactobacillus plantarum* KJ03 showed the best performance in cholesterol removal (53%) [6]. Its beneficial effects in the gut are largely based on its ability to adhere and colonize the intestine. *Lactobacillus plantarum* KCTC10887BP mitigates excessive inflammatory responses in somatic cells, and the key molecule contributing to anti-inflammatory resistance is LTA [7]. Additional *Lactobacillus plantarum* NCU137-fermented *Coix lacryma* not only regulates intestinal flora and the colonic urea nitrogen cycle in mice exposed to high humidity but also significantly enhances immune biochemical markers, restoring immune function [8]. *Lactobacillus plantarum* KU200656, isolated from Korean kimchi, exhibits bacteriostatic effects against *Staphylococcus aureus*, *Listeria monocytogenes*, *Escherichia coli*, and *Salmonella monocytogenes*, and also possesses anti-adhesion properties against pathogenic bacteria [9]. Furthermore, *Lactobacillus plantarum* NCHBL-004 improves glucose metabolism and maintains balanced lipid levels [10].

Key technologies such as strain screening and improvement, high-density fermentation technology, and metabolic pathway analysis and regulation, as well as stability and safety assessments, form the foundation of LAB research and development. Among these, high-density fermentation is a critical step in optimizing LAB production. However, during the high-density fermentation process, several challenges arise, including the deceleration of bacterial growth rates, the accumulation of inhibitory metabolites, and a decline in strain stability. These issues pose significant bottlenecks to the high-quality development of the LAB industry. Consequently, improving the growth density of LABs has become a key focus of research worldwide.

For instance, Choi GH et al. [11] achieved a biomass of 3.845 g/L for the strain *L. Plantarum* 200655, which was 2.429 times higher than that obtained using a non-optimized medium, by conducting a response surface experiment that optimized carbon and nitrogen sources following one-factor-at-a-time screening. Similarly, Gutuierrez-Sarmiento et al. [12] employed response surface optimization to enhance the culture conditions for *Lactobacillus plantarum* BAL-03-ITTG, achieving a viable count of 7.10 × 10^9^ CFU/mL. In another study, Zhang et al. [13] used a Box–Behnken design to optimize the medium components resulting in a viable *Lactobacillus bulgaricus* count of (2.95 ± 0.07) × 10^9^ CFU/mL.

While optimization of nutrient composition and culture conditions is a critical approach for increasing LAB viability, many fermentations still fail to achieve high bacterial density. Addressing these challenges remains a focal point for further advancements in the field. In recent years, researchers have focused on increasing the growth density of bacteria by screening functional oligosaccharides, polysaccharides, protein hydrolysates, and natural plant and herbal extracts. Studies have demonstrated that LABs cultured in media containing bacteriostatic factors can absorb compatible substances that enhance the affinity between water molecules and intracellular biomacromolecules, thereby maintaining protein structure and enzymatic activity, which ultimately increases strain viability [14]. Shakerian et al. [15] found that adding 1% inulin significantly promoted the proliferation of *Bifidobacterium animalis* BB-12 during yogurt fermentation, resulting in a pH of 4.7–4.9. Additionally, Boger et al. [16] observed that *Lactobacillus salivarius* W57 could symbiotically thrive when co-cultured with *Lactobacillus paracasei* W20, attributed to the presence of glycoside hydrolase GH32 in *Lactobacillus paracasei* W20, which enhances its growth in oligofructose. Michael et al. [17] compared the changes in colony count during the low-temperature storage of skim yogurt with and without the addition of plant extracts (olive oil, garlic, citrus, and onion extracts). Their results indicated while plant extracts did not significantly affect *Streptococcus thermophilus* and *Bifidobacterium animalis* in yogurt, they increased the survival rate of *Lactobacillus bulgaricus* and *Lactobacillus acidophilus*. Liu et al. [18] found that the inclusion of arginine and fermentable sugars in the culture medium promoted the growth of *L. buchneri* mycobacteria while studying its metabolic pathways.

Sodium alginate, a natural polysaccharide extracted from brown algae, kelp, or sargassum, possesses a high number of carboxyl and hydroxyl groups, which contribute to its strong chemical reactivity [19,20]. This enables it to quickly form a hydrogel with a three-dimensional cross-linked structure under mild conditions [21]. Sodium alginate is abundant, cost-effective, non-toxic, renewable, and biodegradable, making it an attractive option for various applications [22]. Sodium alginate, as an inert material, hardly interacts with cells, making it ideal for modulating the mechanical properties of the extracellular matrix [23]. However, it is difficult to maintain stable mechanical properties of sodium alginate as a hydrogel for a long period of time. This gelling capability allows for cell encapsulation under physiological conditions, ensuring uniform cell distribution throughout the matrix. Sodium alginate hydrogels are commonly employed as encapsulation materials in cryopreservation due to their stability, biocompatibility, and ability to inhibit ice crystal formation [24,25,26]. Additionally, sodium alginate has demonstrated non-toxic, versatile, and highly biocompatible properties, making it an ideal material for protecting active biological components, particularly for storing probiotics sensitive to heat, pH, and other environmental factors [27,28]. Rodrigues de Freitas et al. preserved zebrafish ovarian tissue by hydrogel-encapsulated cryopreservation, maintaining cell membrane integrity [29]. However, no studies have reported the use of sodium alginate hydrogels in the context of high-density LAB fermentation of LABs.

In this study, *Lactobacillus plantarum* p6, a strain with high resistance to salts and acids isolated in the early stages, was used as the initial organism (Supplementary Figures S3–S5). The media composition, culture conditions, and high-density culture process most suitable for the growth of the strain according to its nutritional requirements were determined. This work aims to establish a robust high-density fermentation process for *Lactobacillus plantarum* P6, providing a foundation for its future development and industrial application.

## 2. Materials and Methods

### 2.1. Strains and Chemicals

The strain used in this study was *Lactobacillus plantarum* P6, isolated from pickled vegetables produced by Sichuan Lao Tan Food Co., Ltd., in Meishan City, Sichuan Province, China. The strain is currently preserved at the Key Laboratory of Comprehensive Utilization of Straw and Blackland Conservation of the Ministry of Education, Jilin Agricultural University.

All other chemicals were analytical grade and commercially available unless otherwise stated. MRS Medium was obtained from Qingdao Hi-Tech Industrial Park Hope BioTechnology Co., Ltd., Qingdao, China; peptone from soya was obtained from Beijing Vokai Biotechnology Co., Ltd., Beijing, China.

### 2.2. Strain Activation

*Lactobacillus plantarum* p6 preserved in glycerol tubes was thawed, and 1 mL was inoculated into an MRS liquid medium, followed by incubation at 37 °C in an anaerobic incubator for 24 h. The activated culture was then transferred to a fresh MRS liquid medium at a 2% inoculum size and subcultured 1–2 times to ensure full activation.

In this study, the incubator used was the SPX-25085H-II incubator, Shanghai Xinmiao, Shanghai, China.

### 2.3. Single-Factor Experiment on Nutritional Conditions of Lactobacillus plantarum P6 Fermentation

The media components most suitable for the growth of *Lactobacillus plantarum* P6 were selected by a single-factor test. In this study, glucose, maltose, lactose, xylose, and sucrose were selected as carbon sources based on the MRS medium, while dried corn syrup powder, beef dip powder, soy peptone, tryptone, and yeast extract powder were chosen as nitrogen sources. The optimal carbon and nitrogen sources, as well as their ideal ratios, were determined based on the growth performance of the strain. Magnesium ions (Mg^2+^) serve as cofactors in most glycolytic pathways and are essential for numerous metabolic processes. So, after screening the optimum Mg^2+^ concentration of *Lactobacillus plantarum* P6 by a single-factor test, other metal ions were added to obtain the best metal ion combination with Mg^2+^. The optimal medium composition for the growth of *Lactobacillus plantarum* p6 was determined based on the viable cell counts after static anaerobic incubation at 37 °C in an anaerobic incubator for 16 h, with a 2% inoculum. The optimum medium for *Lactobacillus plantarum* p6 growth was determined by measuring viable cell counts in these different media combinations.

### 2.4. Single-Factor Experiment on Fermentation Conditions of Lactobacillus plantarum P6

The initial pH and inoculum size most suitable for the growth of *Lactobacillus plantarum* P6 were screened by evaluating single factors. Based on the previous experiments, the initial pH was set at 5, 5.5, 6, 6.5, and 7.0, while the inoculation volume was adjusted to 1%, 2%, 4%, 6%, and 8%, respectively. The optimal growth conditions for *Lactobacillus plantarum* p6 were determined by measuring the viable cell counts after static anaerobic incubation at 37 °C in an anaerobic incubator for 16 h, using a 2% inoculum.

### 2.5. Response Surface Analysis Test

Based on the above experiment by examining the single factors, three factors with more significant effects on the growth of *Lactobacillus plantarum* P6 were selected: maltose concentration, total nitrogen content, and MnSO_4_ concentration. A Box–Behnken response surface optimization test was then performed, using the viable cell counts as the response indicator. The experimental design of the Box–Behnken test is presented in Table 1, and the regression model was analyzed using Design-Expert (v13.0.5.0) software (Table 1 and Table 2).

### 2.6. Effect of Bacteriotropic Factors on Fermentation Culture of Lactobacillus plantarum P6

Most LABs require trace amounts of external vitamins for optimal growth, which play a vital role in facilitating cell proliferation and metabolic activity. In this study, we enriched the fermentation medium with 0.01 g/L of essential vitamins (vitamin C, vitamin H, vitamin PP, vitamin B2, vitamin B1, and pyridoxal hydrochloride) and 0.3 g/L of amino acids (methionine, cysteine salts, cysteine, glycine, glutamic acid, aspartic acid, and histidine). Additionally, 10% vegetable juices (including pumpkin, persimmon, carrot, potato, and cucumber juices) and other enrichment factors were evaluated through the single-factor test.

Sodium alginate is known to enhance cell coagulation and granule formation, and it has abundant hydroxyl and carboxylic acid functional groups which make it an effective adsorbent; changing its concentration gradient (0.1, 0.2, 0.4, 0.6, 0.8, and 1.0%) enables us to investigate its influence on the growth of *Lactobacillus plantarum* p6. The most effective growth-promoting factor was identified based on the viable cell counts, following static anaerobic incubation at 37 °C in an anaerobic incubator for 16 h, with a 2% inoculum.

### 2.7. Effect of Different Incubation Methods on Viable Counts of Lactobacillus plantarum P6 During Fermentation in 5 L Fermenter

In the middle and late stages of fermentation, the sugar content decreases, leading to reduced nutrient availability for the bacteria and an accumulation of metabolic by-products, which alters the fermentation conditions. To maximize the viable cell counts, different neutralizing agents (ammonia and sodium hydroxide) were tested. Once the sugar content dropped below 5 g/L, additional sugar or a complete nutrient medium was continuously fed into the system. The viable cell counts, bacterial wet weight, lactic acid production, and lactic acid concentration were recorded at 2 h intervals. Based on these indicators, the optimal environmental conditions for the growth of *Lactobacillus plantarum* were determined.

### 2.8. Measurement of Viable Cells

The viable cell counts were determined by plate counting method. Based on Gutiérrez-Sarmiento, W et al. [12]. 

The total viable cells with sodium alginate referred to the method by Darjani et al. [30]. Specifically, 1 g of *Lactobacillus plantarum* P6 gel was dissolved in 9 mL of a 0.06 mol/L sodium citrate solution, thoroughly mixed, and shaken at 37 °C ± 1 °C for 40 min. Subsequently, 1 mL of the appropriately diluted sample was plated and incubated, after which colony counts were performed.

Bacterial density was measured using a microplate reader, with 200 μL of the sample added to each well of the microplate. Three parallel tests were conducted, and 200 μL of each sample was used to measure the absorbance value at 600 nm, with the average value taken (FLUOstar Omega Enzyme Labeler, BMG LABTECH, Offenburg, Germany).

## 3. Results and Discussion

### 3.1. Effect of Fermentation Medium on the Viable Counts of Lactobacillus plantarum P6

**Figure 1:**
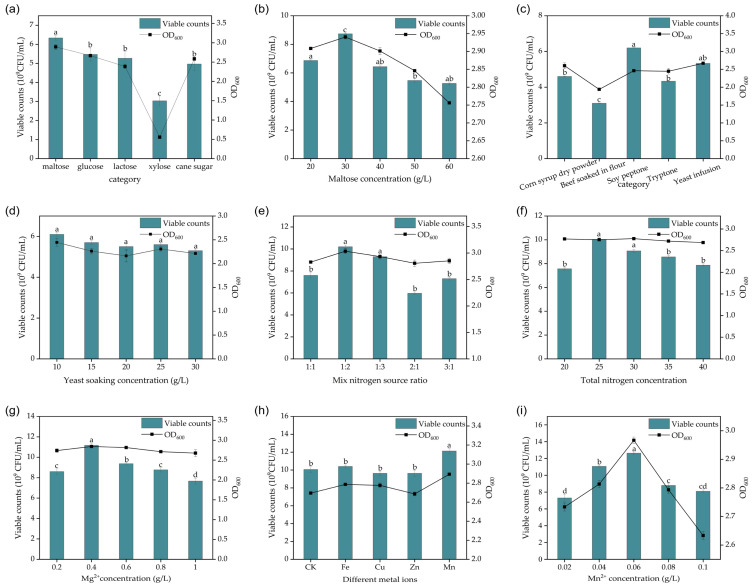
Single-factor analysis of fermentation medium of *Lactobacillus plantarum* p6: (**a**) effect of different carbon sources on viable cell counts during fermentation of *Lactobacillus plantarum* P6; (**b**) effect of maltose concentration on viable cell counts during fermentation of *Lactobacillus plantarum* P6; (**c**) effect of different nitrogen sources on viable cell counts during fermentation of *Lactobacillus plantarum* P6; (**d**) effect of yeast soaking as single nitrogen source on viable cell counts during fermentation of *Lactobacillus plantarum* P6; (**e**) effect of different nitrogen source ratios on viable cell counts of *Lactobacillus plantarum* P6 during fermentation; (**f**) effect of mixed nitrogen source concentration on viable cell counts during fermentation of *Lactobacillus plantarum* P6; (**g**) effect of Mg^2+^ concentration on viable cell counts during fermentation of *Lactobacillus plantarum* P6; (**h**) effect of different metal ions on viable cell counts during fermentation of *Lactobacillus plantarum* P6; (**i**) effect of Mn^2+^ concentration on viable cell counts during fermentation of *Lactobacillus plantarum* P6. Note: Letters a, b, c, and d in figure indicate results of one-way statistical analysis (*p* < 0.05).

Carbon and nitrogen sources serve as the primary energy inputs for microbial growth, influencing microbial metabolic regulation in numerous ways, and are important for microbial growth [31]. Sufficient carbon is particularly vital for optimal microbial growth [32]. When different carbon and nitrogen sources were tested, LABs exhibited varying utilization efficiency. Xylose, a five-carbon sugar, was the least utilized carbon source, likely due to the strain’s weak ability to metabolize pentoses. In contrast, maltose had the most significant effect on bacterial growth. Low sugar concentrations limited bacterial growth due to energy deficiency, while excessively high sugar concentrations potentially induced the production of unwanted metabolites or increased osmotic pressure, causing an inhibitory effect on LABs [33]. The optimal sugar concentration for *Lactobacillus plantarum* P6 growth was 30 g/L, yielding the highest viable cell counts, as shown in Figure 1a,b. Nitrogen is an essential factor in the biosynthesis of nucleic acids or proteins [34]. Regarding nitrogen sources, there was no significant difference in the bacterial utilization of yeast extract and soy peptone. Even at higher concentrations, the viable cell counts remained unchanged when yeast extract was used as a sole nitrogen source. Therefore, a combination of yeast extract and soy peptone was chosen as the nitrogen source for future experiments (Figure 1c,d). The carbon-to-nitrogen ratio(C:N) is critical for microbial growth, as it directly affects both bacterial development and fermentation product accumulation [35]. The highest viable cell count was achieved at a C:N ratio of 1:2 (Figure 1e). Increasing total nitrogen content beyond this ratio led to a decrease in viable counts, likely because at low nitrogen concentrations, growth factors were insufficient, while at higher concentrations, the excessive nitrogen inhibited bacterial growth and metabolism. Mixing nitrogen sources and selecting appropriate nutrient-rich yeast extracts for growth media may be more favorable for bacterial growth [36]. When the total nitrogen source was up to 25 g/L, the viable cell counts reached the maximum (Figure 1f).

Inorganic salts play a crucial role in maintaining microbial cell integrity by stabilizing osmotic pressure, pH, and redox potential, while also influencing enzyme function in various biological processes, all of which are essential for microbial development [37,38]. Different microorganisms respond uniquely to varying types and levels of trace elements. For example, Mg^2+^ not only enhances cellular metabolic activity but also facilitates protein degradation, aiding in the utilization of organic macromolecules. During bacterial growth, ATP synthesis is vital for LAB proliferation, as many transmembrane transport processes and biosynthesis pathways require ATP, which can be promoted by appropriate concentrations of Mg^2+^ [39]. Magnesium ions act as cofactors for key enzymes involved in the growth and metabolism of LABs. Screening for optimal Mg^2+^ levels, based on the viable cell counts, revealed that the addition of 0.4 g/L Mg^2+^ provided the best growth promotion. However, higher concentrations of Mg^2+^ showed an inhibitory effect, likely due to the low Mg^2+^ requirements of this strain (Figure 1g). When combined with other metal ions, the fermentation results followed the order Mn^2+^ > Fe^2+^ > Zn^2+^ > Cu^2+^ in terms of promoting bacterial growth. The combination of Mg^2+^ and Mn^2+^ had the most significant effect on *Lactobacillus plantarum* P6 growth, whereas other combinations showed no notable improvement (Figure 1h). This could be attributed to the fact that Mn^2+^ acts as a cofactor for pyruvate carboxylase and arginine and as an activator of many enzymes, playing a crucial role in the growth and metabolism of LABs [40]. Screening for optimal Mn^2+^ levels based on the viable cell counts revealed that the growth of *Lactobacillus plantarum* P6 was best promoted with the addition of 0.06 g/L Mn^2+^ (Figure 1i).

### 3.2. Effect of Fermentation Culture Conditions on Viable Cell Counts of Lactobacillus plantarum P6

**Figure 2:**
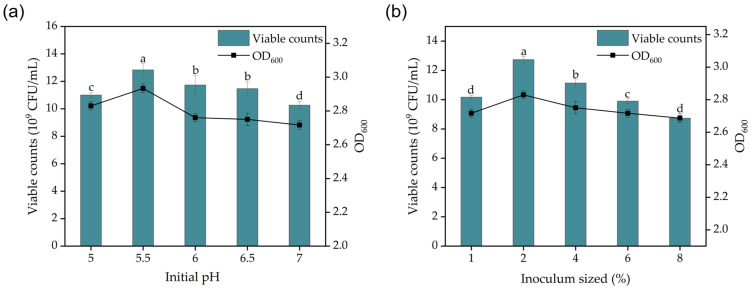
Single-factor analysis of culture conditions for *Lactobacillus plantarum* p6 fermentation: (**a**) effect of different initial pH values on viable cell counts of *Lactobacillus plantarum* P6 during fermentation; (**b**) effect of different inoculum sizes on viable cell counts during fermentation of *Lactobacillus plantarum* P6. Note: Letters a, b, c, and d in figure represent results of one-way statistical analysis (*p* < 0.05).

Microorganisms survive at different initial pH values, and variations in pH values can significantly impact nutrient uptake and utilization efficiency in microbial cells [41]. Too high or too low of a pH can alter the cell membrane and morphology of LABs. During fermentation, LABs produce lactic acid and other acids, which lower the pH of the medium, ultimately inhibiting the bacteria’s growth and reproduction [42]. In low pH environments, lactate exists mainly in an undissociated form and readily penetrates LABs to induce their death [43]. Optimal microbial growth occurs when the pH is within a suitable range, particularly the initial pH [44]. The optimal initial pH for *Lactobacillus plantarum* P6 was determined to be 5.5 based on the highest viable cell counts. Deviations from this pH, either lower or higher, had an inhibitory effect on *Lactobacillus plantarum* p6 (Figure 2a).

Inoculum is one of the most critical parameters in controlling microorganisms in industrial fermentation production. It affects production costs, the duration of the fermentation cycle, fermentation yield, and the risk of microbial contamination. Lower inoculum levels can lead to extended lag phases, prolonged fermentation cycles, reduced yields, and a heightened risk of contamination. Conversely, higher inoculum levels may increase production costs and cause abnormal microbial metabolism, rapid nutrient depletion, and oxygen limitation. Therefore, selecting an appropriate inoculum size is essential to ensure efficient microbial fermentation [45]. For *Lactobacillus plantarum* p6, the highest viable cell counts were achieved with a 2% inoculum level (Figure 2b).

### 3.3. Response Surface Analysis Test Results

**Figure 3:**
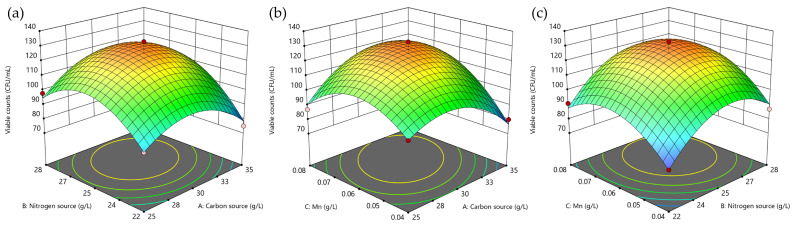
Response surface plots of interaction of factors on viable cell count of *Lactobacillus plantarum* P6: (**a**) AB response surface plots of interaction of factors on viable cell count of *Lactobacillus plantarum* P6; (**b**) AC response surface plots of interaction of factors on viable cell count of *Lactobacillus plantarum* P6; (**c**) BC response surface plots of interaction of factors on viable cell count of *Lactobacillus plantarum* P6. The color change from blue to red corresponds to an increase in responses. The colored circles at the bottom of the image are the projection of the 3D image at the bottom.

Carbon and nitrogen sources play an important role in the growth of microorganisms, essential factors for microbial growth and metabolism; after a single-factor exploration, Mn^2+^ was found to have a significant promotional effect on *Lactobacillus plantarum* P6, so maltose concentration, total nitrogen content, and MnSO_4_ concentration were selected as the three factors of the response surface test, and viable cell counts were taken as the response value to design a three-factor, three-level test (Figure 3). The experimental data were analyzed using Design-Expert software (Table 3). The model’s significance was determined by the *p*-value, with *p* < 0.01 indicating high significance and *p* < 0.05 indicating significance. As shown in Table 3, the *p*-value of the regression equation model is highly significant, and the lack-of-fit term is not significant.

This indicates that the model has a good fit and is reliable. The model’s R^2^ is 0.9758, indicating strong agreement with the actual data, while the Adj-R^2^ value of 0.9446 suggests that 94.46% of the variation in the response can be explained by the model, enabling accurate predictions of the viable cell counts in the medium.

A quadratic regression model was applied to the experimental data to derive the regression equations, assessing the effects of maltose content (*A*), total nitrogen content (*B*), and MnSO_4_ content (*C*) on *Lactobacillus plantarum* P6 viable cell counts (*Y*):

*Y* = +125.4 + 1.38*A* + 9.50*B* + 8.13*C* + 4.75*AB* + 9.50*AB* + 1.25*BC* − 15.70*A^2^* − 18.45*B^2^* − 16.70*C^2^*

The derivation of each of the three variable factors in the equation was performed to determine their maximum values, resulting in a maximum cell density (*Y*) of 1.28 × 10^10^ CFU/mL. At this point, the predicted optimal formulations were identified as maltose 30.89 g/L, total nitrogen content 25.87 g/L, and MnSO_4_ 0.066 g/mL. These results were verified by preparing a culture medium with maltose 31 g/L, yeast extract/soy peptone (1:2) 26 g/L, dipotassium hydrogen phosphate 2 g/L, sodium acetate 5 g/L, diammonium hydrogen citrate 2 g/L, MgSO_4_ 0.4 g/L, and MnSO_4_ 0.07 g/L. Using a 2% inoculum, the culture was incubated at 37 °C in an anaerobic incubator for 16 h, and three parallel validation experiments were conducted. The average value of viable cell counts from these experiments was 1.33 × 10^10^ CFU/mL, representing a 96.24% match with the predicted value, which is a 4-fold increase in viable cell counts compared to unoptimized results (Figure 3).

### 3.4. Effect of Growth-Enhancing Factors on Lactobacillus plantarum P6 Fermentation

LABs have limited biosynthetic capabilities and are vulnerable to various stresses during fermentation, including temperature stress, acid stress, osmotic stress, oxidative stress, and other stresses [46,47,48]. The addition of suitable growth-enhancing factors to the culture medium has been demonstrated to significantly promote the growth of lactic acid bacteria (LABs) [49]. During the fermentation, the gradual accumulation of H^+^ leads to a significant decrease in the pH of the medium, which negatively affects the growth and metabolism of the bacteria as well as lactic acid production. The addition of suitable bacteriostatic substances can help protect the bacteria from environmental stressors, maintaining their viability and activity. Moreover, the inclusion of growth-enhancing substances, such as vitamins, amino acids [50], and vegetable juices [51], has also been found to promote LAB growth.

**Figure 4:**
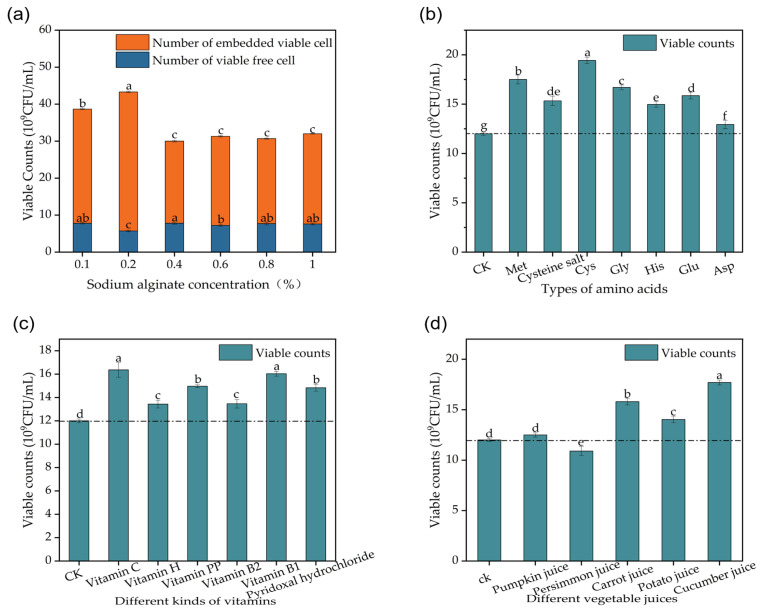
Effect of addition of enrichment substances on viable cell counts during fermentation of *Lactobacillus plantarum* P6: (**a**) effect of varying concentrations of sodium alginate on viable cell counts during fermentation of *Lactobacillus plantarum* P6; (**b**) effect of different amino acids on viable cell counts during fermentation of *Lactobacillus plantarum* P6; (**c**) effect of different vitamins on viable cell counts during fermentation of *Lactobacillus plantarum* P6; (**d**) effect of different vegetable juices on viable cell counts during fermentation of *Lactobacillus plantarum* P6. Note: Letters a, b, c, d, e, f and g in figure show results of one-way statistical analysis (*p* < 0.05).

In experiments where different vitamins, amino acids, and vegetable juices were added, they all promoted the growth of *Lactobacillus plantarum* P6. However, the viable cell counts did not significantly increase to high levels (Figure 4b–d).

Sodium alginate has been shown to be effective in improving nutrient retention [52]. A sodium alginate hydrogel can be used as an adsorbent to effectively adsorb metal cations, etc. Sodium alginate microcapsules are stabilized in gastric acid and also inhibit digestive enzymes when they reach the small intestine and dissolve, reducing cholesterol and glucose absorption and controlling fat digestion [53,54]. There was a significant increase in the total colony count when sodium alginate was added for disintegration treatment, and the total colony count reached 4.3 × 10^10^ CFU/mL at 16 h of fermentation (Figure 4a). Sodium alginate-encapsulated microcapsules showed higher viability than free probiotics under simulated gastrointestinal conditions [55]. The viable cell counts did not increase with the addition of sodium alginate but gradually decreased to remain stable. This probably occurs when too much sodium alginate is added, leading to an increase in the viscosity in the medium, slowing down the conduction speed, and making the solution system unstable.

### 3.5. Effect of Different Culture Conditions on Lactobacillus plantarum P6 Fermentation in 5 L Fermenter

Small-scale fermentation of *Lactobacillus plantarum* P6 was carried out in a 5 L fermenter using the optimum medium composition determined by the single-factor test. A trend graph of sugar content during the fermentation period was generated with a reducing sugar content of the medium and was measured every 2 h. At the 12th h, the maltose concentration in the fermentation broth was 3.4 g/L which decreased to 1.7 g/L by the end of fermentation at the 16th h. Maltose was replenished when its concentration dropped below 5 g/L (Supplementary Figures S1 and S2).

**Figure 5:**
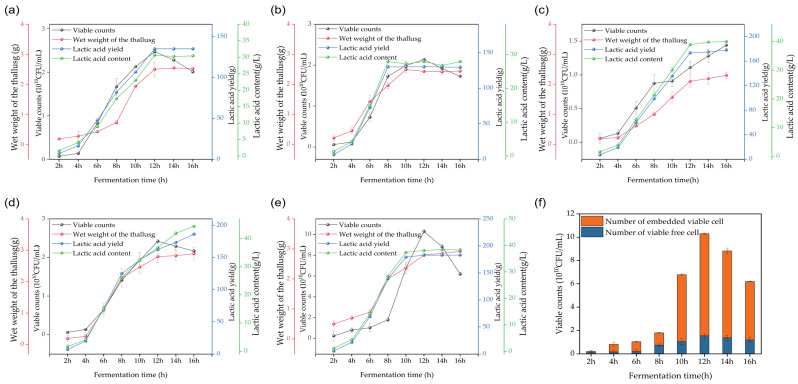
Effect of different culture conditions on viable cell count during the fermentation of *Lactobacillus plantarum* P6 in a 5 L fermenter: (**a**) effect of pH adjustment using sodium hydroxide as a neutralizer in optimal medium formulation on viable cell counts during the fermentation of *Lactobacillus plantarum* P6; (**b**) effect of pH adjustment using ammonia as a neutralizer in the optimal medium formulation on viable cell counts during the fermentation of *Lactobacillus plantarum* P6; (**c**) effect of replenishment of concentrated maltose solution at 12 h of fermentation and by addition of sodium hydroxide as a neutralizer on the viable cell count of fermenting *Lactobacillus plantarum* P6; (**d**) effect of pH adjustment with sodium hydroxide as a neutralizer on viable cell counts of *Lactobacillus plantarum* P6 during fermentation when supplementing the full nutrient medium at 12 h; (**e**) effect of pH adjustment by adding 0.2% sodium alginate and sodium hydroxide as a neutralizer to viable cell counts during the fermentation of *Lactobacillus plantarum P6*; (**f**) effect of sodium alginate addition on the form of *Lactobacillus plantarum* P6 cells present in the fermenter.

In obtaining the highest fermentation density of *Lactobacillus plantarum* P6, based on the results of a single factor to control the pH at 5.5 using ammonia and sodium hydroxide as a neutralizing agent, respectively, with sodium hydroxide as a neutralizing agent in the 12th h, *Lactobacillus plantarum* P6 reached its highest value at 2.47 × 10^10^ CFU/mL (Figure 5a). When ammonia was used as a neutralizer, the viable cell counts reached a peak of 2.14 × 10^10^ CFU/mL at 8 h, followed by a gradual decrease, which shows that ammonium lactate has a greater inhibitory effect on *Lactobacillus plantarum* P6 than sodium lactate (Figure 5b). After determining the optimal neutralizing agent, we chose to replenish the maltose solution and full nutrient medium, respectively, at the 12th h. After replenishing sugar alone, the viable cell counts were not greatly improved but decreased compared to that when sugar was not replenished, which might be due to the excessive sugar content in the medium after replenishment, which inhibited the growth and reproduction of the bacteria (Figure 5c). It is evident that the supplementation of sugar to the medium alone did not have a significant effect on the promotion of *Lactobacillus plantarum* p6. After replenishing the full nutrient medium, the viable cell counts were consistent with the level of viable cell counts in the unsupplemented medium group (Figure 5d), which did not produce an obvious promotion effect, but only slowed down the rate of decline of viable cell counts, which shows that the viable cells in the fermentation broth do not continue to multiply and this is not due to insufficient nutrition. With the addition of 0.2% sodium alginate for the 12th h during fermentation, the total colony count reached 1.02 × 10^11^ CFU/mL, which shortened the fermentation period and also increased the viable cell counts with a significant change of 7.67-fold compared with the commercially available MRS medium. In guessing the possible reasons that sodium alginate in this form may form a protective film, we assume that the experimental strains are protected from the adverse external environment exposure, which is relatively reduced, indirectly reducing the killing of *Lactobacillus plantarum* P6, so that the strains are in a stable environment.

## 4. Conclusions

In this study, the most suitable fermentation conditions for *Lactobacillus plantarum* P6 were obtained by a single-factor examination, under which the viable cell counts were significantly enhanced. Additionally, 0.2% sodium alginate was added as a bacterial enrichment substance. Such composition of the medium permits a high-density fermentation of *Lactobacillus plantarum* P6 (1.03 × 10^11^ CFU/mL) to be achieved, and the bacteria wet weight increased by 2.5-fold and fermentation time reduced to 12 h. The addition of sodium alginate, a bacteriostatic agent, may have protected the bacteria from environmental stresses such as lactate accumulation during fermentation. It likely formed a protective film around the LAB cells, promoting aggregation and enhancing the self-condensation of the strain, potentially triggering bacterial community effects. In this environment, free-living bacteria remained active and continued to proliferate. These findings provide a theoretical basis for the application of *Lactobacillus plantarum* in future industrial production.

## Figures and Tables

**Table 1:** *Lactobacillus plantarum* P6 high-density culture conditions used to optimize the response surface test factors and levels.

Level	Factor		
	(A) Maltose Concentration/g·L^−1^	(B) Total Nitrogen Content/g·L^−1^	(C) MnSO_4_ Concentration/g·L^−1^
1	35	28	0.08
0	30	25	0.06
−1	25	22	0.04

**Table 2:** Box–Behnken experimental design and results.

Run	Factor			
	(A) Maltose Concentration/g·L^−1^	(B) Total Nitrogen Content/g·L^−1^	(C) MnSO_4_ Concentration/g·L^−1^	Response Viable Cell Counts (1 × 10^9^ CFU/mL)
1	0	−1	−1	7.4
2	1	0	0	11.2
3	0	1	1	8.7
4	−1	−1	−1	8.5
5	−1	0	0	8.7
6	1	1	1	10.7
7	0	0	0	12.4
8	0	0	0	13.3
9	−1	0	0	9.3
10	0	0	0	12.3
11	0	0	0	12.2
12	1	−1	0	7.5
13	−1	1	0	9.8
14	0	0	0	12.5
15	0	1	1	10.9
16	1	0	−1	8.0
17	0	−1	1	9.1

**Table 3:** Analysis of variance of the regression equation.

Source	Sum of Squares	Mean Square	F-Value	*p*-Value	
Model	5794.29	643.81	31.31	<0.0001	significant
A—Carbon source	15.13	15.13	0.7355	0.4195	
B—Nitrogen source	722	722	35.11	0.0006	**
C-Mn	528.13	528.13	25.68	0.0015	**
AB	90.25	90.25	4.39	0.0744	
AC	361	361	17.55	0.0041	**
BC	6.25	6.25	0.3039	0.5986	
A^2^	1037.85	1037.85	50.47	0.0002	**
B^2^	1433.27	1433.27	69.7	<0.0001	**
C^2^	1174.27	1174.27	57.1	0.0001	**
Residual	143.95	20.56			
Lack of Fit	66.75	22.25	1.15	0.4302	not significant
Pure Error	77.2	19.3			
Cor Total	5938.24				

Note: “**” indicates a highly significant effect on the results (*p* < 0.01).

## Data Availability

The original contributions presented in the study are included in the article/Supplementary Materials, further inquiries can be directed to the corresponding author.

## Supplementary Materials

The following supporting information can be downloaded at https://www.mdpi.com/article/10.3390/foods13213407/s1: Figure S1: Maltose standard curve; Figure S2: Maltose consumption curve during fermentation in 5 L fermenter; Figure S3: Acid tolerance of *Lactobacillus plantarum* P6; Figure S4: Salt tolerance of *Lactobacillus plantarum* P6; Figure S5: Bile salt tolerance of *Lactobacillus plantarum* P6.
