# Supplementary figures and images for: High-Density Fermentation of Lactobacillus plantarum P6: Enhancing Cell Viability via Sodium Alginate Enrichment
Source: Foods. 2024 Oct 25;13(21):3407. doi: 10.3390/foods13213407 (PMC11545782; doi:10.3390/foods13213407)

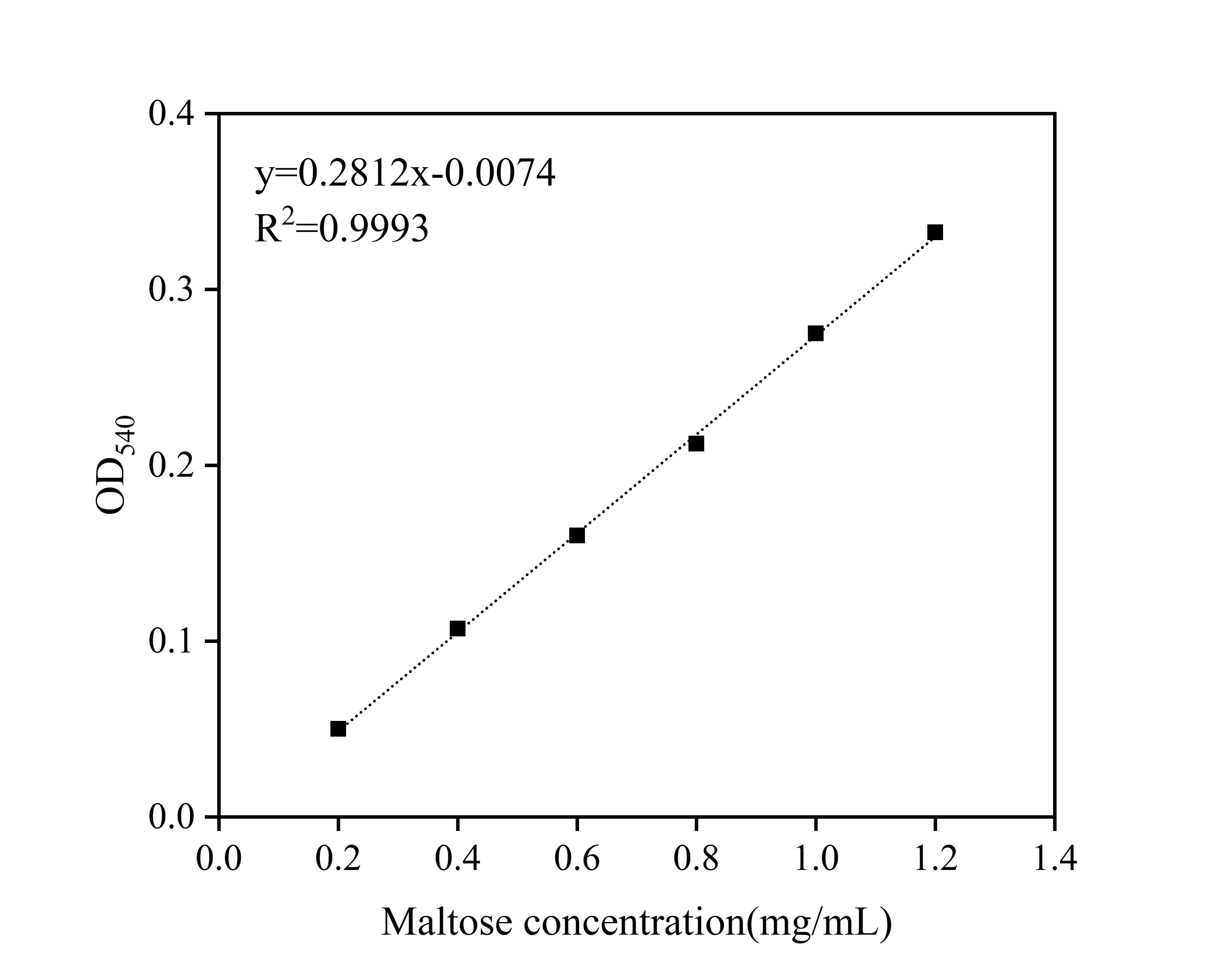

Supplement: Supplementary file 1 [file foods-13-03407-s001.zip › S1 Maltose standard curve.tif]

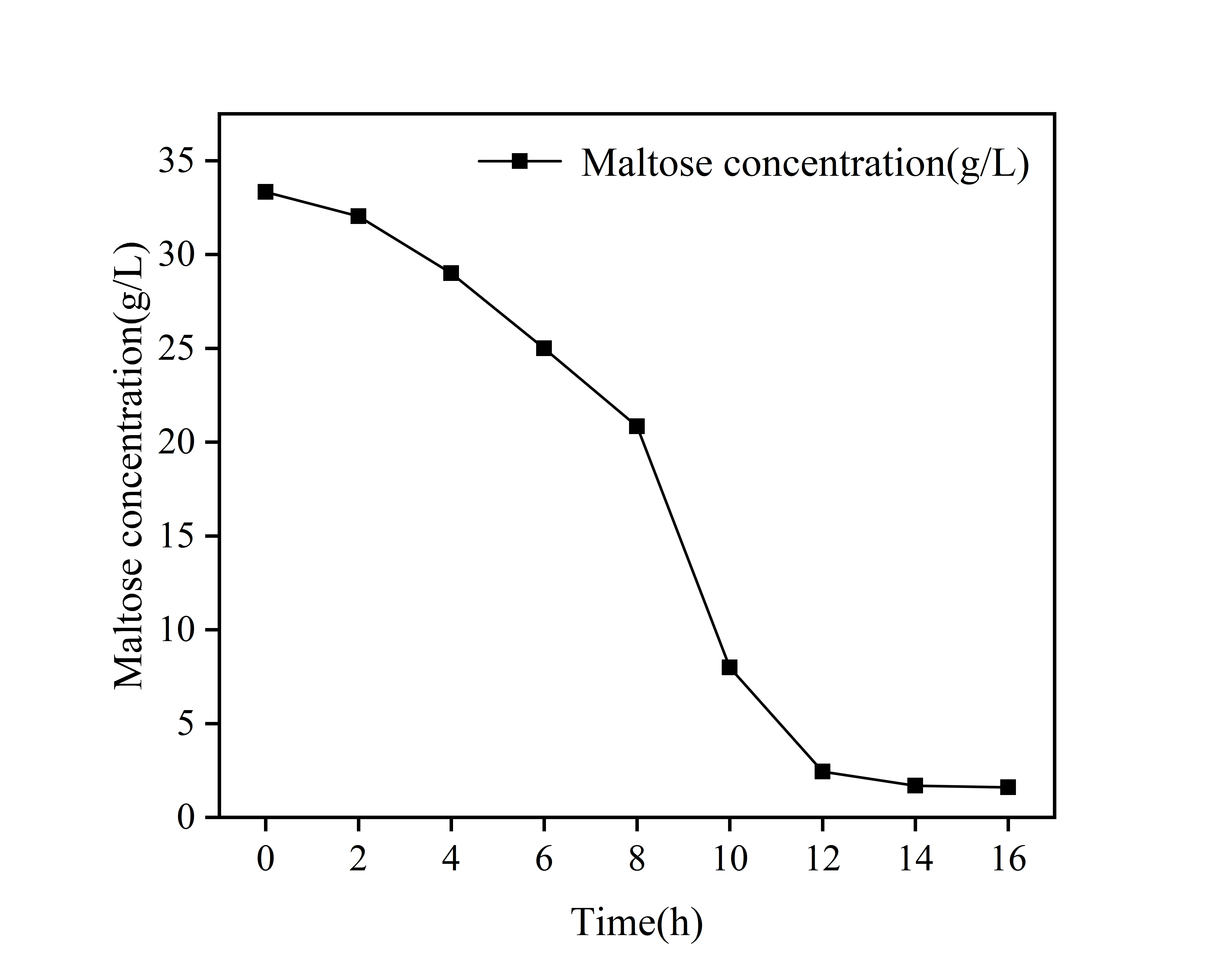

Supplement: Supplementary file 1 [file foods-13-03407-s001.zip › S2 Maltose consumption curve during fermentation in 5L fermenter.tif]

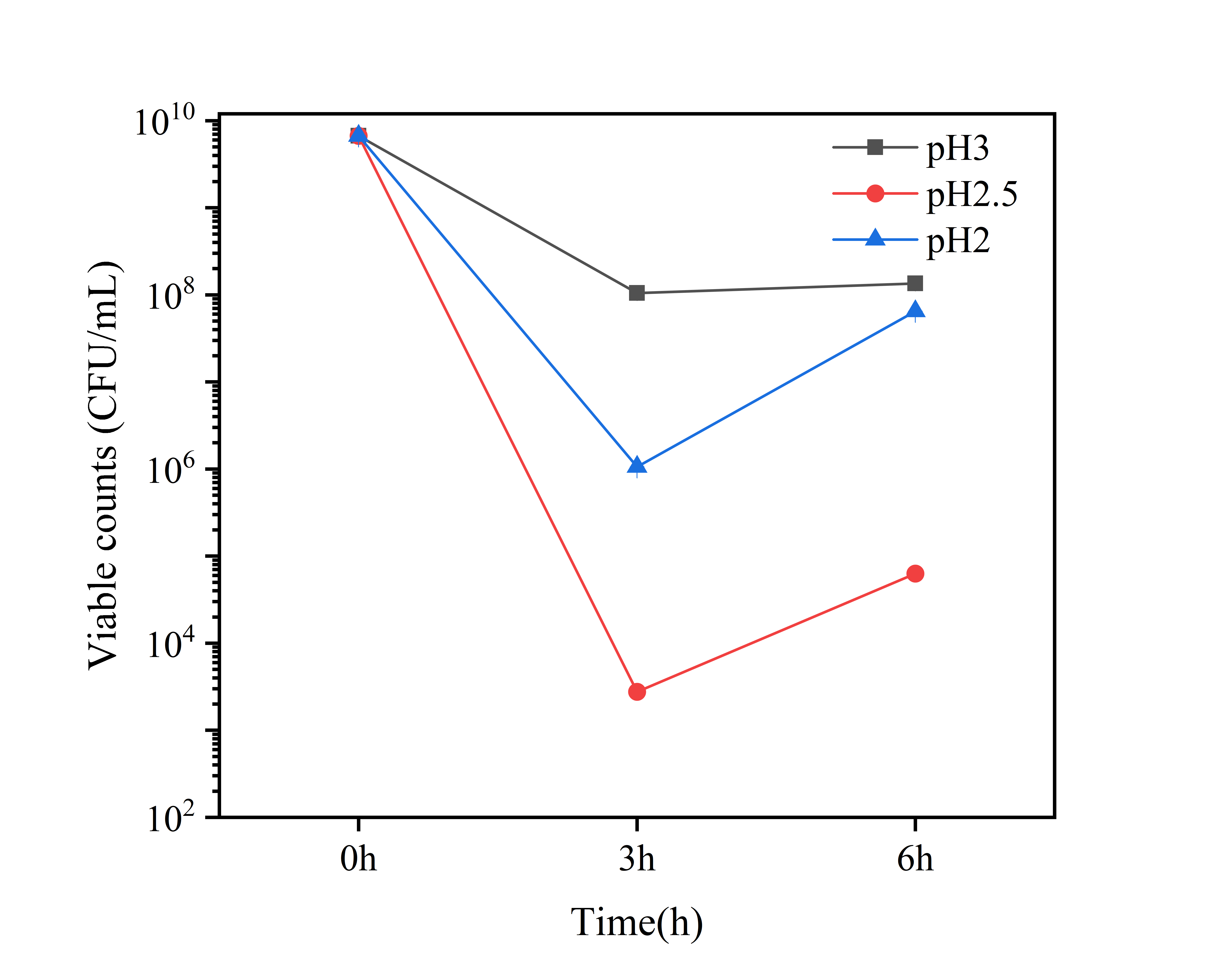

Supplement: Supplementary file 1 [file foods-13-03407-s001.zip › S3 Acid tolerance of Lactobacillus plantarum P6.tif]

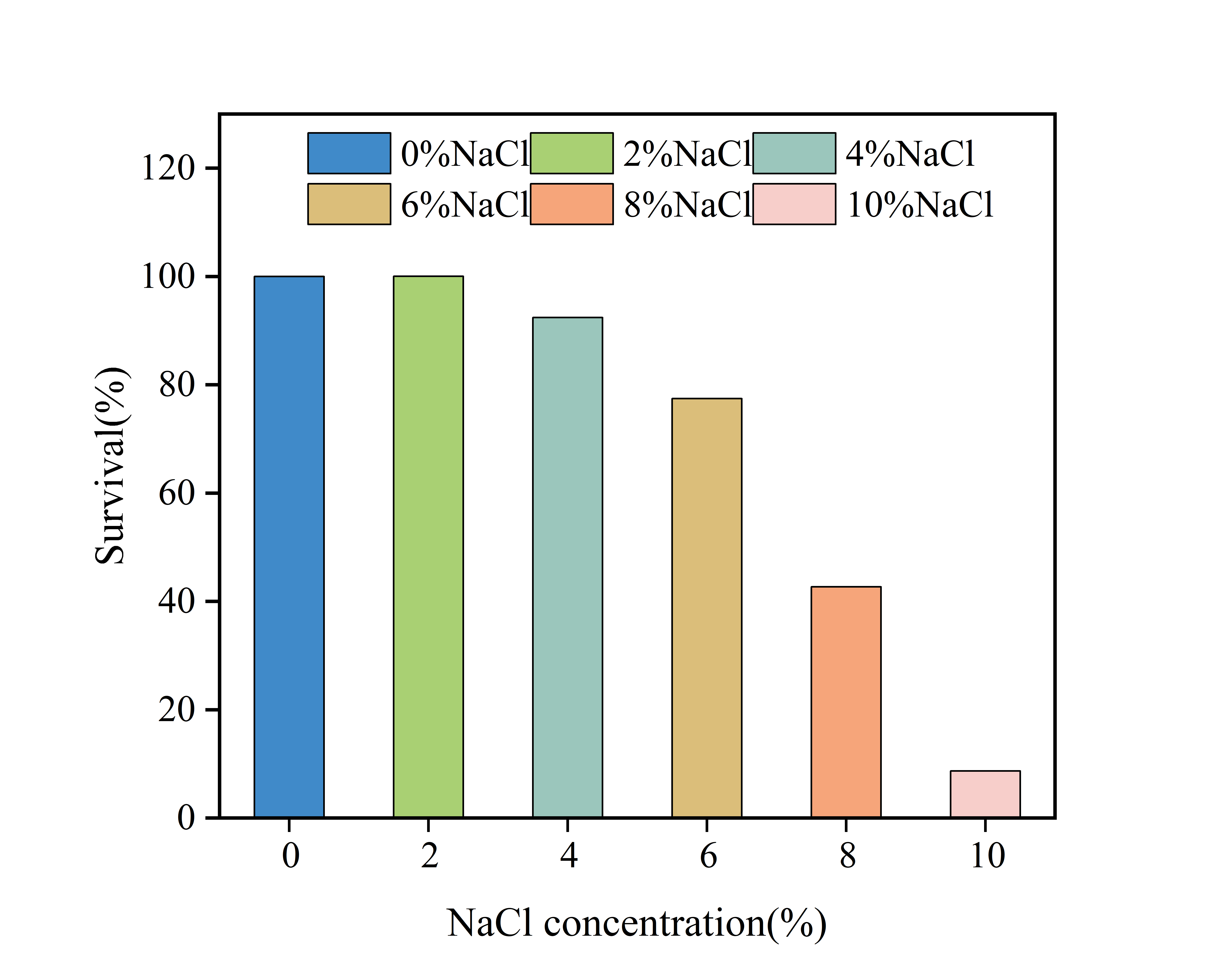

Supplement: Supplementary file 1 [file foods-13-03407-s001.zip › S4 Salt tolerance of Lactobacillus plantarum P6.tif]

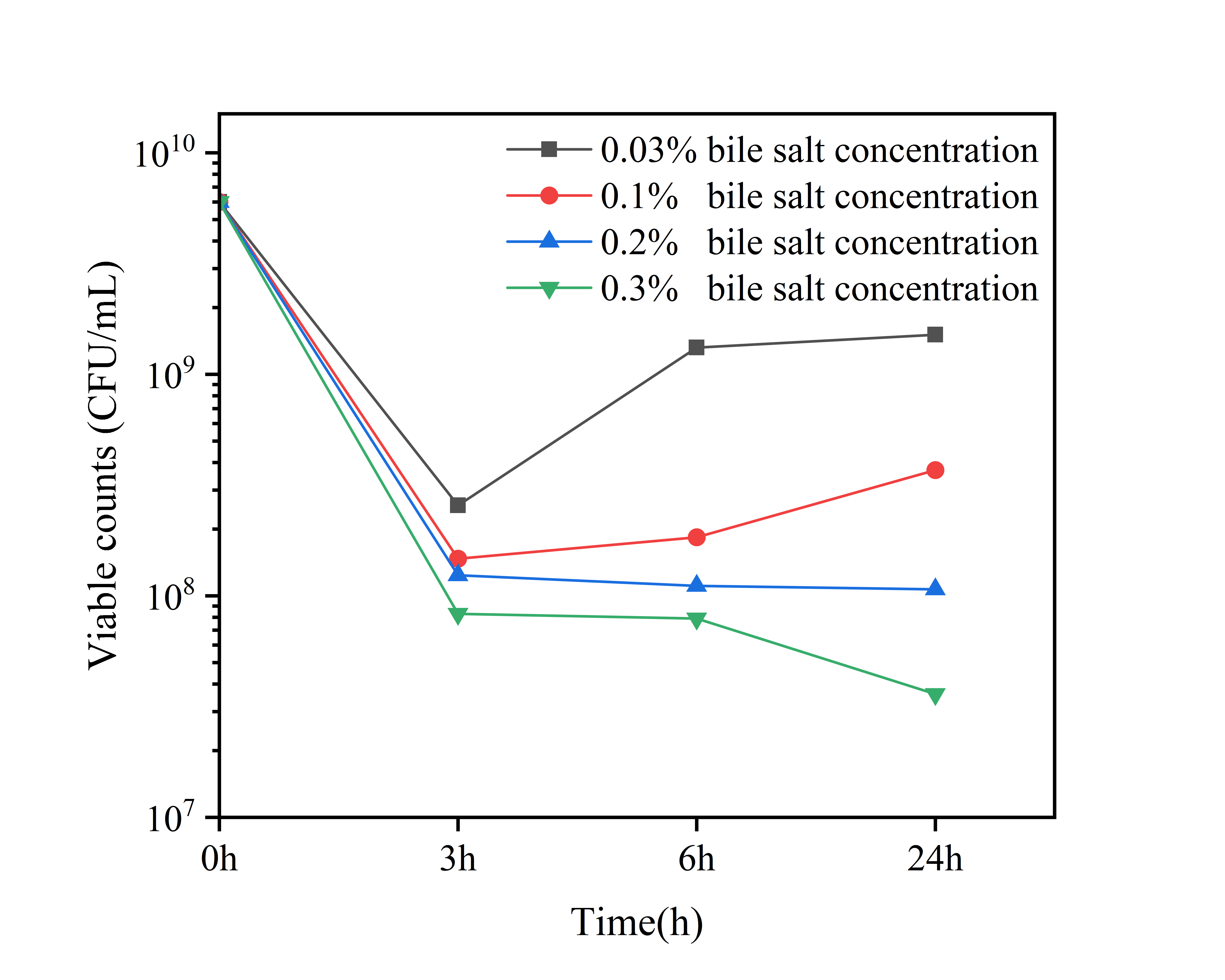

Supplement: Supplementary file 1 [file foods-13-03407-s001.zip › S5 Bile salt tolerance of Lactobacillus plantarum P6.tif]
